# Generation and Characterization of a Tumor Stromal Microenvironment and Analysis of Its Interplay with Breast Cancer Cells: An In Vitro Model to Study Breast Cancer-Associated Fibroblast Inactivation

**DOI:** 10.3390/ijms23126875

**Published:** 2022-06-20

**Authors:** Veronica Romano, Maria Rosaria Ruocco, Pietro Carotenuto, Anna Barbato, Alessandro Venuta, Vittoria Acampora, Sabrina De Lella, Elena Vigliar, Antonino Iaccarino, Giancarlo Troncone, Gaetano Calì, Luigi Insabato, Daniela Russo, Brunella Franco, Stefania Masone, Nunzio Velotti, Antonello Accurso, Tommaso Pellegrino, Giuseppe Fiume, Immacolata Belviso, Stefania Montagnani, Angelica Avagliano, Alessandro Arcucci

**Affiliations:** 1 Department of Public Health, University of Naples Federico II, 80131 Naples, Italy; veronica.romano@unina.it (V.R.); alessandro.venuta@unina.it (A.V.); vit.acampora@studenti.unina.it (V.A.); sab.delella@studenti.unina.it (S.D.L.); elena.vigliar@unina.it (E.V.); antonino.iaccarino@unina.it (A.I.); giancarlo.troncone@unina.it (G.T.); immacolata.belviso@unina.it (I.B.); stefania.montagnani@unina.it (S.M.); 2 Department of Molecular Medicine and Medical Biotechnology, University of Naples Federico II, 80131 Naples, Italy; mariarosaria.ruocco2@unina.it; 3 TIGEM, Telethon Institute of Genetics and Medicine, 80078 Naples, Italy; p.carotenuto@tigem.it (P.C.); a.barbato@tigem.it (A.B.); brunella.franco@unina.it (B.F.); 4 Medical Genetics, Department of Translational Medical Science, University of Naples Federico II, 80131 Naples, Italy; 5 IEOS Istituto di Endocrinologia e Oncologia Sperimentale ‘G. Salvatore’, National Council of Research, 80131 Naples, Italy; g.cali@ieos.cnr.it; 6 Anatomic Pathology Unit, Department of Advanced Biomedical Sciences, School of Medicine, University of Naples Federico II, 80131 Naples, Italy; luigi.insabato@unina.it (L.I.); daniela.russo@unina.it (D.R.); 7 Scuola Superiore Meridionale, School for Advanced Studies, 80138 Naples, Italy; 8 Department of Clinical Medicine and Surgery, University of Naples Federico II, 80131 Naples, Italy; stefania.masone@unina.it; 9 Department of Advanced Biochemical Sciences, University of Naples Federico II, 80131 Naples, Italy; nunzio.velotti@gmail.com; 10 Department of General, Oncological, Bariatric and Endocrine-Metabolic Surgery, University of Naples Federico II, 80131 Naples, Italy; antonello.accurso@unina.it; 11 DAI Chirurgia Generale, Endocrinologia, Ortopedia e Riabilitazione, Azienda Ospedaliera Universitaria Federico II, 80131 Naples, Italy; tommaso.pellegrino@unina.it; 12 Department of Experimental and Clinical Medicine, University “Magna Graecia” of Catanzaro, 88100 Catanzaro, Italy; fiume@unicz.it

**Keywords:** breast cancer, breast cancer-associated fibroblasts, aggregates, breast cancer cells, deactivation, conditioned medium

## Abstract

Breast cancer-associated fibroblasts (BCAFs), the most abundant non-cancer stromal cells of the breast tumor microenvironment (TME), dramatically sustain breast cancer (BC) progression by interacting with BC cells. BCAFs, as well as myofibroblasts, display an up regulation of activation and inflammation markers represented by α-smooth muscle actin (α-SMA) and cyclooxygenase 2 (COX-2). BCAF aggregates have been identified in the peripheral blood of metastatic BC patients. We generated an in vitro stromal model consisting of human primary BCAFs grown as monolayers or 3D cell aggregates, namely spheroids and reverted BCAFs, obtained from BCAF spheroids reverted to 2D cell adhesion growth after 216 h of 3D culture. We firstly evaluated the state of activation and inflammation and the mesenchymal status of the BCAF monolayers, BCAF spheroids and reverted BCAFs. Then, we analyzed the MCF-7 cell viability and migration following treatment with conditioned media from the different BCAF cultures. After 216 h of 3D culture, the BCAFs acquired an inactivated phenotype, associated with a significant reduction in α-SMA and COX-2 protein expression. The deactivation of the BCAF spheroids at 216 h was further confirmed by the cytostatic effect exerted by their conditioned medium on MCF-7 cells. Interestingly, the reverted BCAFs also retained a less activated phenotype as indicated by α-SMA protein expression reduction. Furthermore, the reverted BCAFs exhibited a reduced pro-tumor phenotype as indicated by the anti-migratory effect exerted by their conditioned medium on MCF-7 cells. The deactivation of BCAFs without drug treatment is possible and leads to a reduced capability of BCAFs to sustain BC progression in vitro. Consequently, this study could be a starting point to develop new therapeutic strategies targeting BCAFs and their interactions with cancer cells.

## 1. Introduction

Breast cancers (BCs), the most frequent cancers in women, are very heterogeneous solid tumors [1] that can be subdivided into distinct histological and molecular subtypes, linked to different invasive capabilities, sites of metastasis and clinical outcomes [2,3]. In particular, ductal and lobular BCs represent the most common histological types of invasive BCs [4]. Ductal BCs originate from mammary gland ducts, whereas lobular BCs originate from the lobules of the mammary gland [5]. Furthermore, BCs can be classified into three molecular subtypes depending on the expression of the estrogen receptor (ER), progesterone receptor (PR) and human epidermal growth factor receptor 2 (HER-2). Triple negative breast cancers (TNBCs) lacking the expression of the three receptors are associated with the worst prognosis [1,6]. Solid tumors, such as BCs, are constituted by heterogeneous tissues containing cancer cells and an abnormal and dynamic tumor microenvironment (TME), influencing tumor development and dissemination [3,7,8,9]. The TME consists of tissue-specific resident and recruited non-cancer stromal cell types, such as cancer-associated fibroblasts (CAFs), immune cells, endothelial cells, pericytes, adipocytes, mesenchymal stem cells (MSCs), blood vessels, lymphatic tumor vessels and the extracellular matrix (ECM) [1,2,3,10,11,12]. In particular, breast cancer-associated fibroblasts (BCAFs) are the most abundant cell type among non-cancer stromal cells of the breast TME. By interacting with cancer cells, BCAFs contribute to BC growth and metastatic dissemination, which is the most important cause of BC-connected deaths [1,2]. BCAFs sustain cancer cell migration and invasion by secreting soluble factors such as TGF-β, HGF, basic fibroblast growth factor (bFGF), FSP-1, CCL11, CXCL14, CCL-2 and IL-6. Moreover, IL-32 and IL-6 secreted by BACFs induce a cascade of molecular mechanisms associated with metastasis [2].

Over 80% of stromal fibroblasts in BC acquire the activated phenotype of myofibroblasts, which are activated fibroblasts involved in wound healing and inflammatory processes [13]. Therefore, BCAFs exhibit characteristics similar to those of myofibroblasts, such as the up regulation of activation and inflammation markers represented by α-smooth muscle actin (α-SMA) and cyclooxygenase 2 (COX-2) [2,14,15]. However, unlike myofibroblasts, BCAFs remain in the tumor stroma even if the activated stimuli are reduced [1]. Additionally, CAFs have been described as dangerous travel companions due to their ability to form homotypic and heterotypic cellular aggregates with circulating cancer cells [16]. Circulating cancer cells alone hardly establish secondary tumor growth due to anoikis, literally meaning “without home”, which occurs when tumor cells leave the stromal microenvironment of the primary tumor mass [16]. Conversely, CAFs can facilitate the survival and colonization in distant body sites of cancer cells by disseminating through the blood circulation as circulating CAFs or CAF aggregates, with or without cancer cells [17,18,19]. Circulating CAFs and CAF aggregates have been identified in the peripheral blood of patients with metastatic diseases in breast, lung and prostate cancers. These circulating CAFs and CAF aggregates are promising biomarkers for cancer progression and metastatic disease [20,21,22]. However, their role in cancer growth remains poorly analyzed and requires further validations and investigations. The use of tri-dimensional (3D) cell culture systems, such as spheroids, represents a valuable in vitro tool to study the biology and behavior of cancer and non-cancer cells, as well as CAFs, growing as 3D cellular aggregates [23,24,25]. In addition, it has been widely recognized that spheroid models possess many hallmarks commonly found in solid tumors [26]. Consequently, 3D cultures are currently used in a broad range of biological studies, including tumor biology [27], cell adhesion and migration, epithelial morphogenesis, tumor–stroma crosstalk [28], drug screening and resistance, as well as the development of bio-artificial tissues [29,30,31,32]. In our previous work, we demonstrated that during 3D cell culture, normal human cutaneous myofibroblasts, grown as spheroids, undergo a deactivation process associated with a dramatic down-regulation of both α-SMA and COX-2 protein expression levels [33]. Furthermore, we also showed that the cells of spheroids, reverted to adhesion growth after 216 h of 3D cell culture, are proto-myofibroblast-like cells which retain an inactivated phenotype in culture, trigger cancer cell death and dramatically inhibit cancer cell migration by paracrine interactions [14]. Therefore, in this work we have developed and characterized an in vitro stromal experimental system constituted by BCAFs grown as monolayers, BCAFs grown as spheroids and so-called reverted BCAFs. Specifically, BCAF monolayers were isolated from invasive breast carcinomas, expressing both the ER and PR. BCAF spheroids, from human primary BCAFs, were collected and analyzed after 72 h and 216 h of 3D cell culture, and reverted BCAFs were obtained from BCAF spheroids reverted to adhesion growth after 216 h of 3D culture. Moreover, we analyzed the paracrine interactions between this stromal cell type and MCF-7 BC cells focusing on the influence of the conditioned media from the BCAF populations on the viability and migration of BC cells. This experimental system could lead to a better understanding of the role of BCAFs growing as monolayers and aggregates on BC cell viability and migration. In addition, this in vitro model can provide the possibility to study and develop a new therapeutic strategy targeting the TME.

## 2. Results

### 2.1. Characterization of Fibroblasts from Normal and Cancer Breast Tissues

**Figure 1:**
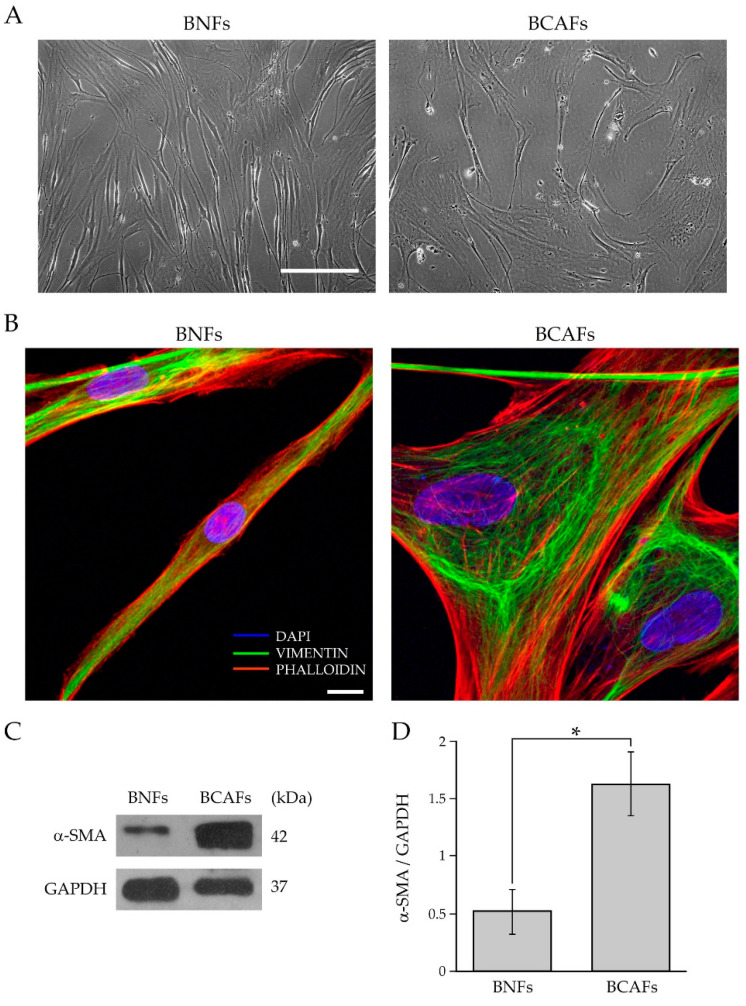
Characterization of fibroblasts isolated from normal and cancerous human breast tissues. (**A**) Representative cell morphology of BNFs and BCAFs by phase-contrast microscopy. Scale bar 200 µm. Magnification ×10. (**B**) Analysis of the cytoskeletal organization of BNFs and BCAFs by confocal fluorescence microscopy. Vimentin immunofluorescence (green channel) and phalloidin staining (red channel) of representative BNFs and BCAFs. DAPI (blue channel) was used to locate the nuclei. Images correspond to the 3D reconstruction of Z planes acquired from the top to the bottom of cells. Scale bar 10 µm. The images are representative of three independent experiments. (**C**) Western blotting analysis of α-SMA in protein extracts of BNFs and BCAFs; GAPDH was used as the loading control. A representative image of three independent experiments is shown. (**D**) Densitometric analyses of α-SMA protein levels. Data are reported as means of at least three independent experiments ± S.E.* *p* < 0.05.

We first characterized fibroblasts isolated from surgically resected breast tumors and compared their morphology to fibroblasts isolated from healthy breast tissues. As shown in Figure 1A, the primary fibroblasts from the normal and cancer tissues displayed the typical spindle-like shape.

However, the fibroblasts from the normal breast tissues (BNFs) exhibited a thinner, elongated and regular spindle-shaped morphology. Conversely, the primary fibroblasts from the tumor tissue, i.e., BCAFs, had a large and irregular spindle-shaped or stellate-like morphology. These results are consistent with several studies showing that CAFs and normal fibroblasts (NFs) display a different cell morphology [1,34,35,36]. Furthermore, human primary BCAFs and BNFs were analyzed by confocal and western blot analysis, regarding the cytoskeleton organization and α-SMA protein levels. It is well recognized that CAFs and NFs differ in their cytoskeleton organization [36] and α-SMA expression levels [36,37]. Therefore, to analyze the cytoskeleton organization of fibroblasts from normal and cancer tissues, we evaluated the presence of intermediate filaments and stress fibers by vimentin immunofluorescence and phalloidin fluorescence staining, respectively (Figure 1B). The confocal immunofluorescence analysis showed a different pattern of stress fibers in the primary BCAFs and BNFs. In particular, BCAFs displayed more evident and well-developed stress fibers. Conversely, BNFs exhibited fewer, less evident and less organized stress fibers. The vimentin immunofluorescence showed the presence of mesenchymal intermediate filaments in both BCAFs and BNFs, thus confirming their mesenchymal origin. CAFs are described as fibroblasts in a permanent state of activation. The most widely used CAF marker is α-SMA [13], which reflects the fibroblast activation state [14,38] and is more expressed in CAFs than NFs [37]. Of note, fibroblasts in different states of activation are known to affect cancer growth differently. Furthermore, increased α-SMA expression in CAFs correlates with tumor aggressiveness and with a poor overall survival rate in BC patients [39]. Therefore, to evaluate the state of activation of the BCAFs and BNFs, we analyzed α-SMA protein levels in extracts of the primary BNFs and BCAFs by western blotting analysis (Figure 1C). The densitometric analysis showed that BCAFs expressed significantly higher protein levels of α-SMA than BNFs (Figure 1D).

**Figure 2:**
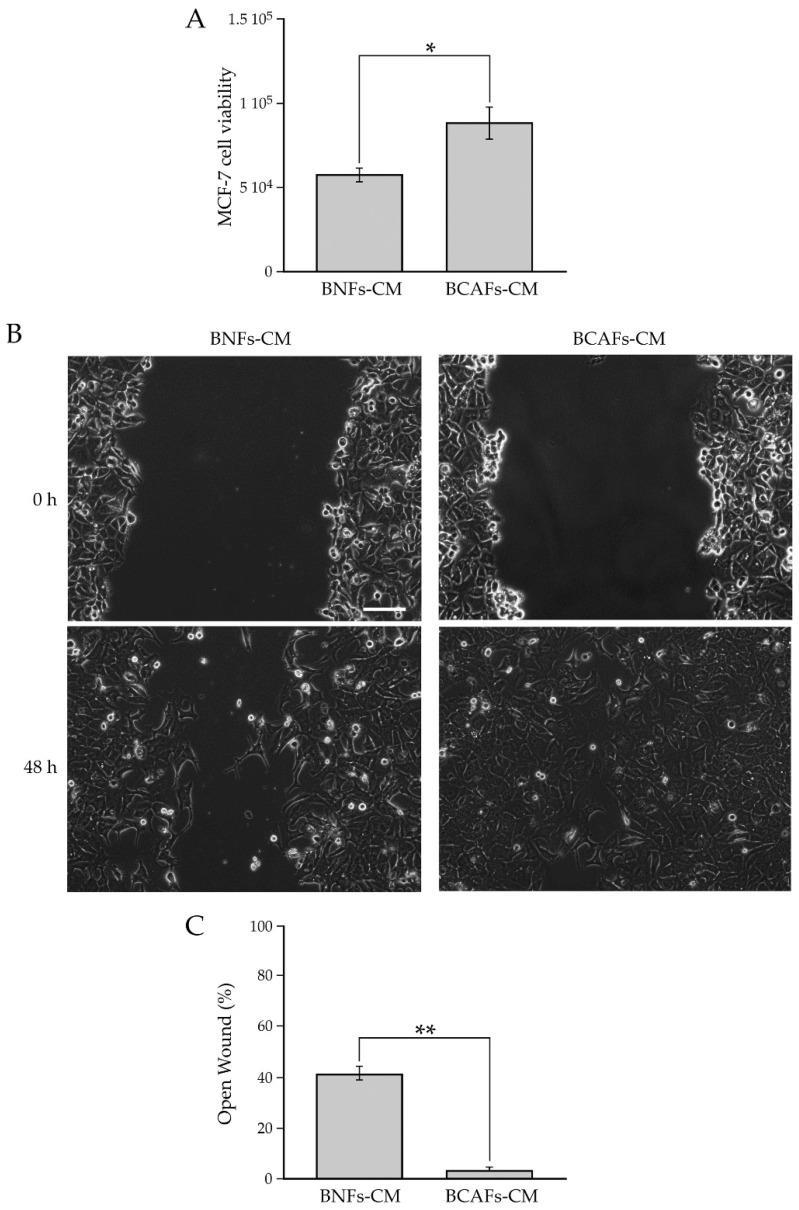
(**A**) Influence of conditioned media from BNFs (BNFs-CM) and BCAFs (BCAFs-CM) on MCF-7 cell viability. Data are means of at least three independent experiments ± S.E. * *p* < 0.05. (**B**,**C**) Influence of conditioned media from BNFs (BNFs-CM) and BCAFs (BCAFs-CM) on MCF-7 cell migration. (**B**) Wound-Healing Assay performed on MCF-7 cells treated with fibroblast-conditioned media. Representative images of three independent experiments show the same fields with scratching at 0 and 48 h after wounding. Scale bar 100 µm. Magnification ×10. (**C)** Migratory capability quantification of MCF-7 cells. Wound widths were measured at 0 and 48 h after wounding. Data are expressed as percentage of fold decrease in open wound area compared with control (0 h) set as 100% and are reported as mean of three independent experiments ± S.E. ** *p* < 0.0001.

In addition, we performed a functional characterization of BNFs and BCAFs by testing the effect of their conditioned media on the proliferation and migratory capability of the MCF-7 breast cancer cell line (Figure 2).

Our analysis showed that the conditioned medium from BCAFs significantly increased the viability of MCF-7 cells compared with the conditioned medium from BNFs (Figure 2A). We further demonstrated that BCAF-derived conditioned medium significantly increased MCF-7 cell migration compared with BNF-conditioned medium (Figure 2B). In particular, 48 h after wounding, the quantitative analysis (Figure 2C) showed a percentage of open surface area of about 40% in MCF-7 cells incubated with BNF-conditioned medium, while the scratch area was almost closed in MCF-7 cells treated with BCAF-conditioned medium. Taken together, all these data indicated that we successfully isolated human primary BCAFs with an activated and pro-tumor state.

**Figure 3:**
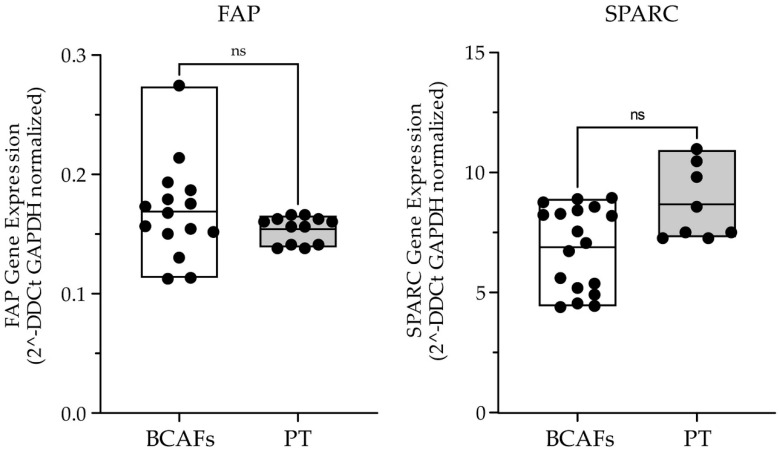
Real-Time Expression analysis of the CAF markers FAP and SPARC in human primary BCAFs (N = 3) and BC patient tissues (PT) (N = 3). Data are shown as percentage of mean +/− SD from triplicates of three independent experiments. Statistical analyses were conducted by one-way ANOVA with Tukey’s post hoc analysis. ns: not significant.

Then, we analyzed, with a Real-Time PCR, the expression of two important CAF markers, such as FAP and SPARC (or osteonectin) [1], in primary BCAFs and paraffin-embedded breast cancer tissues (Figure 3).

The analysis showed no significant difference between the in vitro primary BCAFs and breast cancer tissues for both FAP and SPARC. Therefore, our in vitro BCAFs can be considered as a valuable and helpful in vitro model that could mimic the in vivo breast TME.

### 2.2. Analysis of Human Primary BCAFs Grown as Monolayers or Spheroids

**Figure 4:**
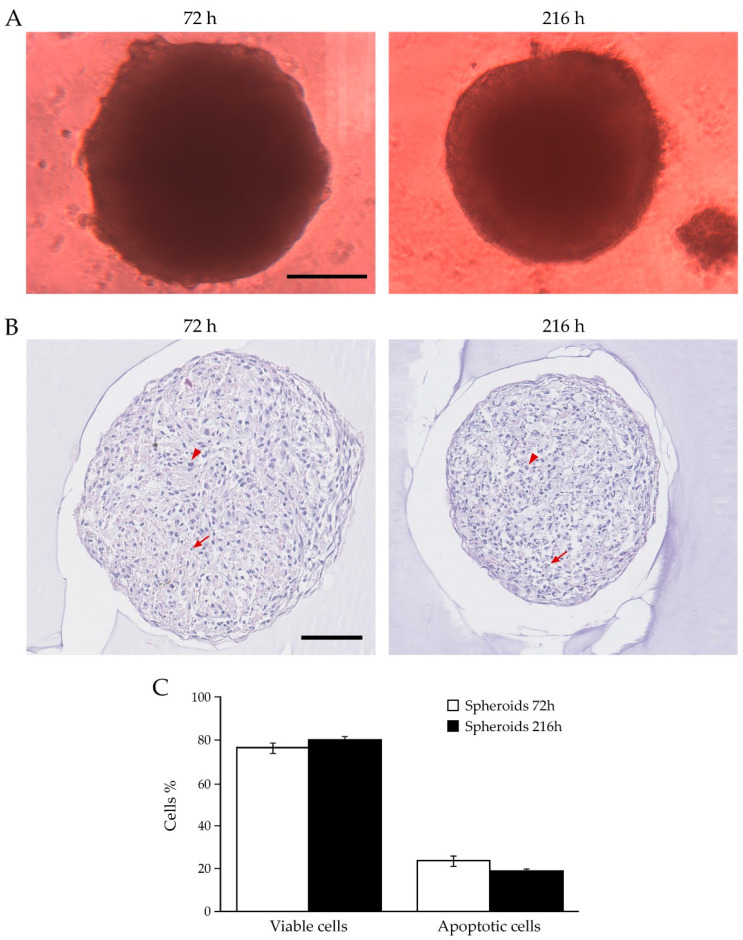
Analysis of BCAFs grown as spheroids. (**A**) Representative images of BCAF spheroids at 72 h and 216 h by phase-contrast microscopy. Scale bar 200 μm. Magnification ×10. Volumes of spheroids collected at 72 and 216 h were 0.109 mm^3^ ± 0.0075 and 0.0864 ± 0.0057, respectively. Data are reported as means of at least three independent experiments ± S.E. *p* < 0.05. (**B**) Hematoxylin and eosin staining of paraffin-embedded sections of BCAF spheroids collected at 72 and 216 h of 3D cell culture. Arrowheads indicate viable cells, while arrows indicate apoptotic cells. Scale bar 100 μm. Magnification ×20. (**C**) Live and apoptotic cell graph of spheroids collected at 72 h and 216 h.

We have previously demonstrated that during spheroid formation, normal primary human myofibroblasts undergo a deactivation process, associated with a significant reduction in α-SMA and COX-2 protein expression levels [14,33]. Furthermore, the fibroblasts of spheroids, reverted to adhesion and monolayer growth, retain in the culture an inactivated phenotype [14]. This is in agreement with other studies showing that myofibroblasts are not terminally differentiated cells and can return to a less activated or quiescent state [40,41,42]. Conversely, CAFs are permanently active fibroblasts that are unable to come back to a less activated, quiescent or anti-tumor state in vivo without drug treatments [1,43]. BCAFs, as well as CAFs from the other solid tumors, are cells in a constitutively activated state [1] associated with high levels of α-SMA and COX-2 proteins. Furthermore, stromal α-SMA and COX-2 expression is linked to BC aggressiveness and invasiveness [44,45]. Therefore, we investigated the molecular and functional profiles of human primary BCAFs isolated from BC patients and grown in vitro as cell monolayers in 2D cultures or aggregates, such as spheroids, in 3D cell cultures. We first generated spheroids of BCAFs upon 72 h and 216 h of a 3D cell culture on agar. Morphology analysis by phase-contrast microscopy showed that BCAF spheroids at both time points had a spherical and regular shape with smooth edges, especially in the spheroids collected at 216 h (Figure 4A).

**Figure 5:**
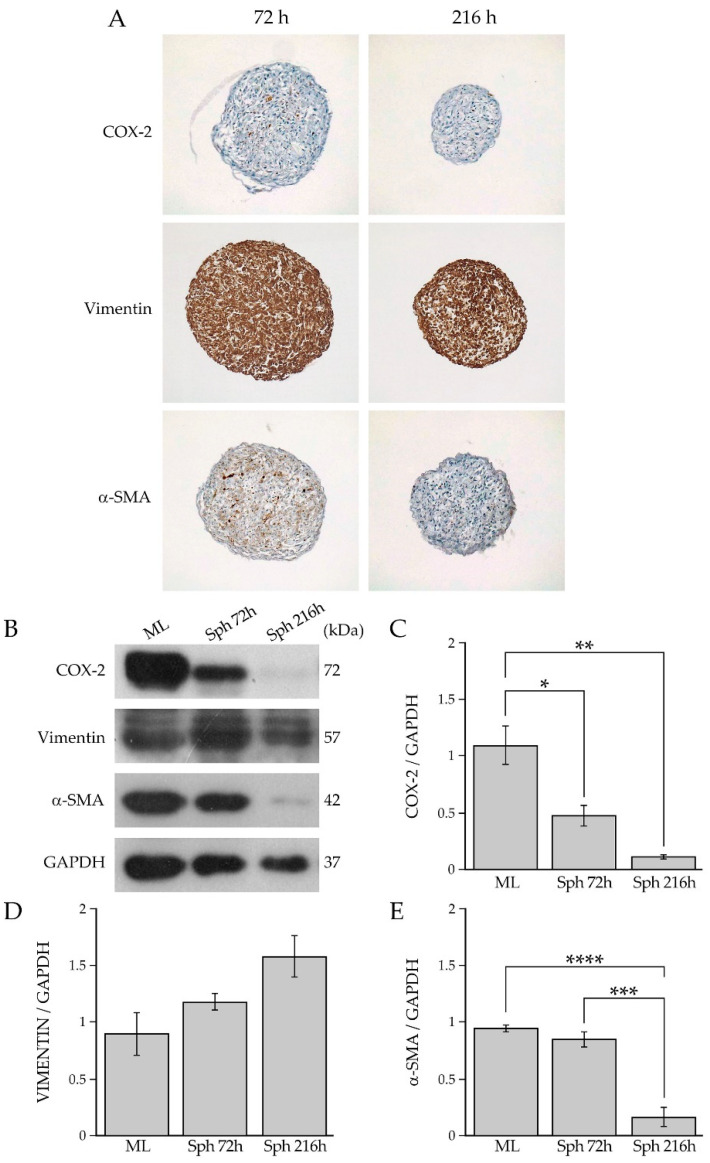
(**A**) Immunohistochemical analysis of COX-2, vimentin and α-SMA in BCAF spheroids collected at 72 h and 26 h. Magnification ×10. (**B**) Western blotting analysis of COX-2, vimentin, and α-SMA in protein extracts of BCAF monolayers (ML) and BCAF spheroids collected at 72 h (SPH 72 h) and 216 h (SPH 216 h) of 3D cell culture on agar. GAPDH was used as the loading control. Representative images of three independent experiments are shown. Densitometric analyses of (**C**) COX-2, (**D**) vimentin and (**E**) α-SMA protein levels. Data are reported as means of three independent experiments ± S.E. * *p* < 0.05, ** *p* < 0.005, *** *p* < 0.001, **** *p* < 0.0005.

The microscopic evaluation of the hematoxylin and eosin-stained sections of the spheroids at 72 h and 216 h showed a prevalence of viable cells and the presence of scattered apoptotic cells. This investigation revealed that there were no significant differences between the percentage of apoptotic and viable cells within the spheroids collected at both time points. (Figure 4B,C). Furthermore, COX-2, vimentin, which is a marker of mesenchymal cells, and α-SMA immunohistochemical analysis was performed on the paraffin-embedded sections of the BCAF sections collected at 72 h and 216 h (Figure 5A).

The analysis showed a weak-to-absent level of staining for COX-2 in the spheroids at 72 h and 216 h, respectively. A moderate immunostaining for α-SMA was detected in BCAF spheroids at 72 h, while a weak immunostaining for α-SMA was detected in the spheroids at 216 h. Immunostaining for COX-2 and α-SMA did not reveal any preferential expression sites. Positive cells for COX-2 and α-SMA were scattered in the spheroids (Figure 5A). Additionally, the immunohistochemical analysis showed strong and homogeneous immunostaining for vimentin in both the spheroids collected at 72 h and 216 h (Figure 5A). Then, to evaluate and compare the state of activation and inflammation of BCAFs grown in vitro as either 2D cell cultures or 3D cell aggregates, we performed a western blotting analysis of COX-2 and α-SMA in protein extracts of BCAF monolayers and BCAF spheroids collected after 72 h and 216 h of 3D cell cultures (Figure 5B). This analysis showed that BCAF monolayers expressed both α-SMA and COX-2 proteins (Figure 5B), confirming their myofibroblast and inflammatory phenotype, which are typical features of CAFs [1,37]. No significant differences were detected in α-SMA protein levels between BCAF monolayers and BCAF spheroids at 72 h (Figure 5B,E). Conversely, a dramatic and significant decrease in α-SMA protein levels was observed in BCAF spheroids collected at 216 h with respect to both BCAF monolayers and BCAF spheroids at 72 h (Figure 5B,E). Hence, the α-SMA protein levels decreased in a time-dependent manner during BCAF aggregation. Interestingly, a massive and significant reduction in the COX-2 protein levels was detected in BCAF spheroids at 72 h compared with BCAF monolayers (Figure 5B,C). This protein reduction was much more evident in BCAF spheroids at 216 h (Figure 5B,C). Hence, the dramatic decrease in the myofibroblast marker α-SMA and the pro-inflammatory protein COX-2 in BCAF spheroids collected at 216 h suggests that during cluster formation, BCAFs gradually acquire a deactivated and less inflammatory phenotype. As previously mentioned, while several studies claim that myofibroblasts are flexibly activated and deactivated in response to changes due to tissue alterations and wound healing [14,33,46,47,48], CAFs are still described as permanently active fibroblast-like cells unable to return to a quiescent state or tumor-suppressive state without pharmacological interventions [1,43]. To the best of our knowledge, there are numerous agents targeting the biological functions of CAFs in vitro and some of them were assessed by clinical trials [49]. Our study demonstrated that not only normal myofibroblasts but also CAFs have a more flexible phenotype by switching from an activated to a less active state without being drug targeted in response to changes in the surrounding environment and growth conditions. In particular, our analyses revealed that BCAF spheroids collected at 72 h exhibited a myofibroblastic and less inflammatory phenotype, whereas BCAF spheroids collected at 216 h displayed a much more deactivated state, characterized by a non-myofibroblastic and less inflammatory phenotype. The analysis of the mesenchymal marker vimentin, by western blotting, did not show any significant differences among the samples (Figure 5B,D). Hence, our data indicated that during the in vitro cell aggregation, BCAFs lost their constitutively activated phenotype by decreasing α-SMA protein expression in a time-dependent manner and reduced their pro-inflammatory phenotype by decreasing COX-2 protein expression.

### 2.3. Reversion of BCAF Spheroids to Adhesion and Monolayer Growth

**Figure 6:**
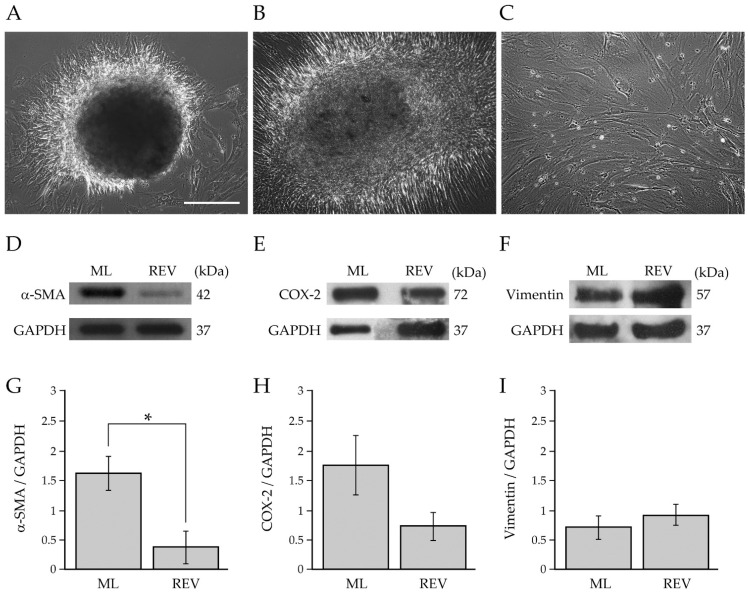
Characterization of reverted BCAFs. (**A**,**B**) Representative images of the outgrowth and spreading of reverted BCAFs from BCAF spheroids transferred on a conventional 2D cell culture dish to allow 2D cell growth and proliferation after 216 h of 3D cell culture on agar. Reverted BCAFs outgrowing from spheroid after (**A**) 1 day and (**B**) 1 week of 2D cell growth. (**C**) Representative cell morphology of reverted BCAFs by phase-contrast microscopy. Scale bar 200 µm. Magnification ×10. (**D**–**I**) Western blotting analysis of α-SMA, COX-2 and vimentin in protein extracts of BCAF monolayers (ML) and reverted BCAFs (REV). GAPDH was used as loading control. Representative images of at least three independent experiments are shown. (**G**–**I**) Densitometric analyses of α-SMA, COX-2 and vimentin protein levels. Data are reported as means of at least three independent experiments ± S.E. * *p* < 0.05.

The study of fibroblast activation/deactivation processes is of fundamental importance for understanding the role of BCAFs in BC growth and progression. In our previous works, we demonstrated that normal primary human myofibroblasts are not terminally differentiated cells and can return to a less activated state in vitro [14,33]. We further demonstrated that these deactivated fibroblasts exert a stronger anti-tumor activity than normal myofibroblasts in vitro [14,33]. Based on this experimental evidence, we evaluated the reversibility of the deactivated and less inflammatory phenotype acquired by BCAFs during 3D cell culture by analyzing the so-called reverted BCAFs, which were generated from BCAF spheroids collected after 216 h of 3D culture on agar. Precisely, we obtained the reverted BCAFs by transferring and culturing BCAF spheroids on conventional 2D cell culture dishes. The high stiffness and flat surface of the cell plastic dishes allowed for the spheroids’ adhesion and the consequent outgrowth and spreading of BCAFs from the spheroids (Figure 6A,B).

The reverted BCAFs were expanded and maintained as a 2D cell culture for about 20–30 days to allow the full outgrowth of BCAFs from spheroids (Figure 6C). As shown in Figure 6C, the reverted BCAFs displayed a large and irregular spindle-shaped or stellate-like morphology. To evaluate the state of activation and inflammation of reverted BCAFs, we performed a western blotting analysis of protein extracts from BCAF monolayers and reverted BCAFs. The protein expression level of α-SMA was diminished dramatically and significantly in reverted BCAFs compared to BCAF monolayers (Figure 6D,G). Reverted BCAFs also showed a decrease, although not significant, in COX-2 protein levels (Figure 6E,H). Our results indicate that during the transition from a 3D to 2D cell culture, reverted BCAFs partially retained the inactivated phenotype of spheroids. No significant differences were detected in the vimentin protein expression levels, confirming the mesenchymal phenotype of both BCAF monolayers and reverted BCAFs (Figure 6F,I).

### 2.4. Evaluation of BC Cell Viability after Paracrine Interaction with BCAFs Grown as Monolayers, Spheroids or Reverted

**Figure 7:**
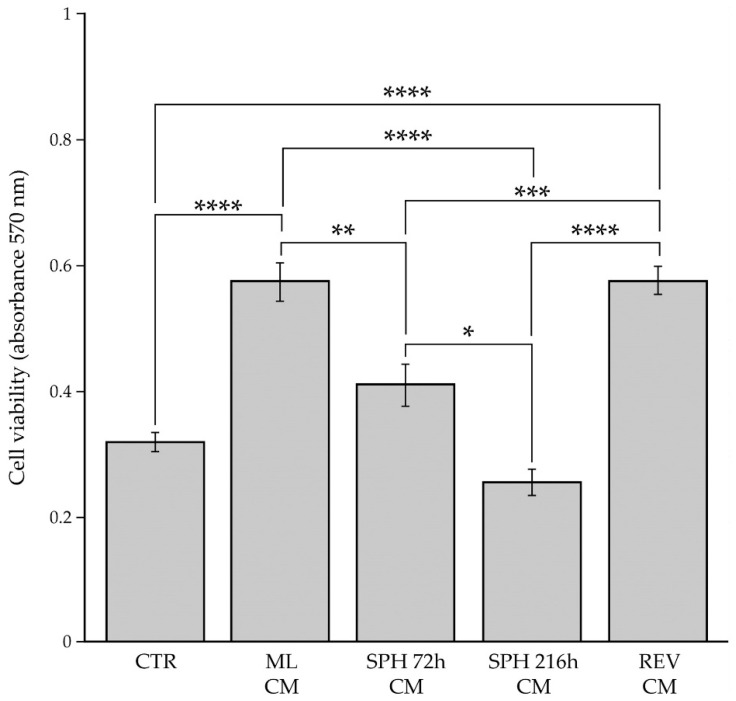
Influence of tumor stromal microenvironment on the viability of BC cells. MCF-7 cells were grown for 48 h with the conditioned media derived from BCAF monolayers (ML CM), spheroids at 72 h (SPH 72 h CM), spheroids at 216 h (SPH 216 h CM) or reverted BCAFs (REV CM). Control is represented by MCF-7 cells incubated with unconditioned medium (CTR). The cell viability was evaluated by MTT assay. Data are means of three independent experiments ± S.E. * *p* < 0.005, ** *p* < 0.001, *** *p* < 0.0005, **** *p* < 0.0001.

BCAFs have been shown to promote BC progression by sustaining, through paracrine interactions, the viability of tumor cells in the primary tumor mass and in the metastatic niche of a secondary tumor [1,2,50,51]. In the primary and secondary tumor sites, BCAFs generate permissive and suitable microenvironments for cancer cell survival and proliferation through the activation of signaling pathways common to both physiological and pathological cellular functions [2,52,53]. Furthermore, during the metastatic dissemination, BCAFs can be found as circulating BCAF aggregates in the bloodstream, probably en route between the primary tumor and sites of metastasis to influence cancer cell seeding and growth in a secondary tumor [2,19,20]. Based on this evidence, our study has also aimed to investigate the paracrine influence of BCAF monolayers, BCAF spheroids and reverted BCAFs on BC cell viability. Therefore, we performed the MTT assay on MCF-7 BC cell line to monitor cell viability following 48 h of treatment with the conditioned media derived from BCAF monolayers, BCAF spheroids at 72 and 216 h and conditioned medium derived from reverted BCAFs (Figure 7).

We used MCF-7 cells incubated with unconditioned cell culture medium (Figure 7) as the control. Our results revealed that BCAF monolayer-conditioned medium significantly increased MCF-7 cell viability compared with the control medium, thus confirming the crucial role of BCAFs in promoting BC growth. However, the survival advantage conferred by BCAF monolayers was lost during BCAF aggregation. In fact, a significant decrease in cell viability was detected in MCF-7 cells treated with the conditioned media derived from BCAF spheroids at both time points compared with the same cells exposed to BCAF monolayer-conditioned medium. Interestingly, the conditioned medium from BCAF spheroids collected at 216 h, which acquired a non-myofibroblastic and less inflammatory phenotype, significantly reduced MCF-7 cell viability with respect to the conditioned medium from BCAF spheroids collected at 72 h, which exhibited a myofibroblastic but less inflammatory phenotype as indicated by the high levels of the α-SMA protein and low levels of the COX-2 protein. No significant differences were observed between MCF-7 cells treated with the control unconditioned medium or the conditioned media derived from BCAF spheroids at 72 h and 216 h. Therefore, BCAF spheroids at both time points did not alter the viability of MCF-7 cells when compared with the cells under the standard cell culture conditions (control). Interestingly, the conditioned medium from reverted BCAFs, as well as the conditioned medium from BCAF monolayers, significantly increased BC cell viability compared with both the control and conditioned media from BCAF spheroids at 72 h and 216 h. Furthermore, the conditioned media of reverted BCAFs and BCAF monolayers affected MCF-7 cell viability to the same extent. Therefore, our results revealed that the reversion of BCAF spheroids to 2D cell growth allowed BCAFs to restore their initial capability to increase MCF-7 cell viability.

### 2.5. Evaluation of BC Cell Migration after Paracrine Interaction with BCAF Monolayers and Reverted BCAFs

**Figure 8:**
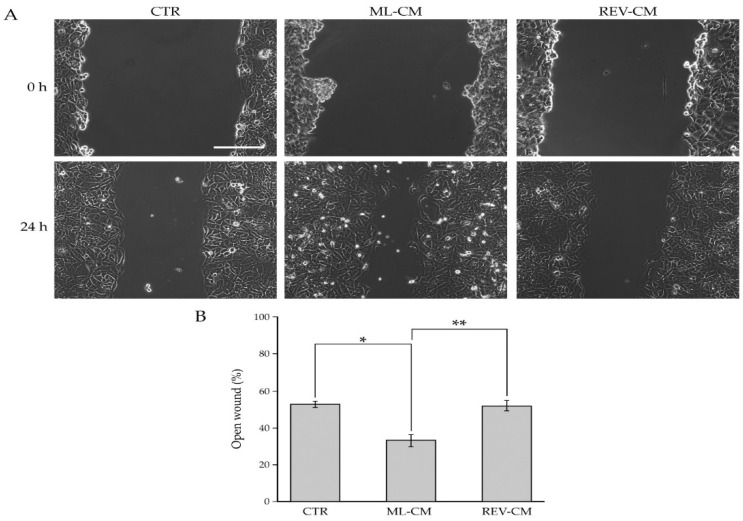
Influence of BCAF monolayers and reverted BCAFs on the migratory capability of BC cells. (**A**) Wound-Healing Assay of MCF-7 cell line grown for 24 h with the conditioned media from BCAF monolayers (ML CM) and reverted BCAFs (REV CM). Standard cell culture medium was used as control. The representative images of three independent experiments show the same fields with scratching at 0 and 24 h after wounding. Scale bar 200 µm. Magnification ×10. (**B**) Migratory capability quantification of MCF-7 cells. Wound widths were measured at 0 and 24 h after wounding. Data are expressed as percentage of fold decrease in open wound area compared with control (0 h) set as 100% and are reported as mean of three independent experiments ± S.E. * *p* < 0.0005, ** *p* < 0.0001.

It is well known that BCAFs establish a fine-tuned relationship with cancer cells and sustain their migration through the release of many secreted factors [2,54]. To evaluate the influence of BCAF monolayers and reverted BCAFs on BC cell migration, we performed Wound-Healing Assays on MCF-7 cells treated with conditioned media derived from BCAF monolayers and reverted BCAFs. A standard cell culture medium was used as the control (Figure 8).

This analysis showed that BCAF monolayer-conditioned medium significantly increased MCF-7 cell migration compared with the control medium, thus confirming the pro-migratory role of BCAFs on cancer cells. On the other hand, unlike BCAF monolayer-conditioned medium, the conditioned medium from reverted BCAFs failed to promote BC cell migration. Indeed, no differences were detected between MCF-7 cells treated with reverted BCAF-conditioned medium and the control unconditioned medium. Of special note is the fact that the migratory capability of MCF-7 cells treated with reverted BCAF-conditioned medium was significantly decreased compared with that of MCF-7 cells treated with the BCAF monolayer-conditioned medium. In particular, 24 h after wounding, the quantitative analysis (Figure 8B) showed a percentage of open surface area in MCF-7 cells incubated with the control medium of about 52 %. Conversely, at the same time point, in MCF-7 cultures treated with the conditioned media of BCAF monolayers and reverted BCAFs, the percentage of open wound areas was about 33% and 51%, respectively.

## 3. Discussion

The study of the reversibility of the myofibroblast phenotype is particularly important for the treatment of diseases, such as solid tumors including BCs, which involve myofibroblasts that in vivo do not revert to a normal phenotype and thus remain constitutively activated. In our previous works, we demonstrated that the deactivation of normal human myofibroblasts, forced to grow as spheroids, is associated with an enhanced capability to impair cancer cell viability and migration in vitro [14,33]. BCAFs preserve a constitutively myofibroblastic phenotype and play a fundamental role in tumor cell proliferation and migration in both primary and secondary tumors [1,2,55]. Interestingly, although CAFs are described as constitutively activated cells, several works demonstrated that it is possible to normalize CAFs or inhibit their biological functions through the administration of specific CAF-targeting agents, antibodies targeting CAF-surface markers or existing compounds that also have a strong influence on CAF functions [49,56,57,58,59,60]. Therefore, targeting CAFs has become an increasingly appealing approach for cancer therapies. Furthermore, the detection of BCAF aggregates in the peripheral blood of patients with metastatic BC leads one to hypothesize that BCAF aggregates could represent markers of BC metastasis and could influence the metastatic process [20].

In this work, we have demonstrated that human primary BCAFs preserve a flexible phenotype by switching from an active to a less active state without being genetically manipulated or drug treated in vitro. In particular, we demonstrated that during spheroid formation, BCAFs undergo a step-by-step deactivation process and that BCAF monolayers and reverted BCAFs play different roles in the regulation of cancer cell migration. We showed that after 216 h of suspension growth as spheroids, BCAFs acquire an inactivated phenotype associated with a dramatic reduction in both α-SMA and COX-2 protein levels. The inactivation of BCAFs within spheroids was also supported by the MTT assay, which showed a significant decrease in the viability of MCF-7 cells incubated with the conditioned medium derived from BCAF spheroids, mainly from the spheroids at 216 h, compared with MCF-7 cells incubated with the BCAF monolayer-conditioned medium. These results strongly support BCAF deactivation as a potential approach to prevent BC progression through the reduction in BC cell viability. Furthermore, these data demonstrated that BCAFs are not cells in a permanent state of activation but they can change their phenotype and reacquire a less activated and less tumorigenic phenotype in response to changes of growth conditions. Therefore, the analysis of the BCAF spheroid-conditioned medium could lead to the identification of soluble factors or vesicles hindering BC cell viability. Furthermore, our data are in agreement with our previous works showing that the influence of fibroblast paracrine signals on cancer cell viability depends on the activation state of the fibroblasts. Indeed, we have previously demonstrated that the conditioned medium from inactivated fibroblastic cells significantly reduces cancer cell viability compared with the conditioned medium derived from myofibroblasts [14,33].

It is noteworthy that reverted BCAFs displayed a different phenotype compared with BCAF monolayers, although the conditioned media of these two cell types equally influenced MCF-7 cell viability. In fact, reverted BCAFs showed a significant and dramatic decrease in α-SMA levels with respect to BCAF monolayers. Reverted BCAFs further exhibited a reduction, although non-significant, in COX-2 protein expression levels. This is the first time that BCAFs have been reprogrammed towards a less activated cell type, associated with a dramatic reduction of α-SMA without exogenous molecules or drug treatment. α-SMA is a marker linked to CAF activation and to a score of cancer progression [31].

Based on different levels of α-SMA, two major subpopulations of CAFs have been identified in solid tumors: myofibroblastic CAFs characterized by high α-SMA expression levels and inflammatory CAFs with a high expression of tumor-promoting cytokines and chemokines [61]. Furthermore, it was reported that BCAFs with a high α-SMA expression are preferentially detected in more aggressive breast tumors, whereas less activated BCAFs showing a very low or no α-SMA expression are preferentially found in less aggressive BCs [62]. In our experimental system, the significant decrease of α-SMA in reverted BCAFs is associated with the anti-migratory activity of their conditioned medium on MCF-7 cancer cells. These results indicate that reverted BCAFs are less activated fibroblasts compared with BCAF monolayers [20]. Moreover, it is noteworthy that FAP and SPARC gene expression analysis in primary BCAFs and paraffin-embedded BC tissues showed that our in vitro tumor stromal microenvironment could be comparable to in vivo tumor stromal microenvironment.

During the last years, important advances have been made in understanding the mechanisms underlying the influence of BCAFs on cancer cell migration associated with metastatic dissemination. Metastasis is the leading cause of BC-related deaths [2]. Therefore, an improved comprehension of the processes regulating cancer cell migration could provide new prognostic biomarkers and therapeutic strategies to both prolong survival and improve the quality life of patients with metastatic BC. BCAFs significantly sustain BC metastasis [1,63]. In particular, BCAFs growing as adherent cells interact with cancer cells by paracrine interactions, remodel the TME and sustain migration and the metastatic capability of neoplastic cells [2]. In our experimental system, further studies will be performed to identify the processes regulating the biochemical and functional rewiring of reverted BCAFs and to determine the differences between conditioned media derived from BCAF monolayers, BCAF spheroids and reverted BCAFs. These analyses could justify the cytostatic and anti-migratory effects of the conditioned media from BCAF spheroids and reverted BCAFs, respectively.

This study highlights the molecular and functional plasticity of primary BCAFs in response to different cell culture conditions. Moreover, our results showed the reversibility of BCAF-activated phenotype without pharmacological treatment and thus led to the hypothesis that the in vitro generation of BCAF aggregates and reverted BCAFs could represent an in vitro strategy to induce BCAF inactivation. However, further studies are required to better understand the biological meaning and eventually identify the mechanisms leading to the functional and biochemical rewiring undergone by reverted BCAFs in vitro. Finally, in our experimental system, the characterization of factors regulating the interactions between stromal and cancer cells could be useful to study and develop new therapeutic strategies hindering the interplay between fibroblasts and cancer cells.

## 4. Materials and Methods

### 4.1. Isolation and Culture of Primary Breast Fibroblasts

Normal breast tissues were obtained from 3 patients undergoing reduction mammoplasty and BC specimens were obtained from patients with ER+/PR+ invasive BCs (n = 8, all females). Primary BNFs and BCAFs were isolated as previously described. Briefly, fresh normal and cancerous breast tissues were cut into smaller pieces (1–2 mm), and after repeated PBS washing, were plated and cultured in Dulbecco’s minimal essential medium (DMEM; Sigma-Aldrich, Saint Louis, MO, USA) supplemented with penicillin (100 mg/mL), streptomycin (100 mg/mL) and 20% fetal bovine serum (FBS, GIBCO, Grand Island, NY, USA) at 37 °C in the presence of 5% CO2. The outgrowth of primary fibroblasts from surgical fragments was observed after about 1 week. The patient-derived fibroblasts sequentially outnumbered the cancer cells, which are known to be sensitive to sequential passaging. Indeed, epithelial cells are known to disappear after passage three [64]. All the experiments were performed only with cells from an early passage (<10). For clinical information of all the utilized breast tumors, refer to Table 1.

### 4.2. Immunofluorescence and Confocal Microscopy for Human Primary Breast Fibroblast Characterization

A morphological analysis of the BNFs and BCAFs was performed as previously described [14,33]. In particular, fibroblasts were plated on glass coverslips fixed with 4% paraformaldehyde, permeabilized with 0.1% Triton X-100, blocked in donkey serum (Millipore, Billerica, MA, USA) and diluted to 1:10 in 1× PBS for 30 min at room temperature. The glass coverslips were incubated with a mouse monoclonal anti-vimentin primary antibody (Sigma-Aldrich), diluted to 1:50 for 1 h at 37 °C, washed three times with 1× PBS and then subsequently incubated with an FITC donkey, anti-mouse secondary antibody (Jackson ImmunoResearch, Suffolk, UK) diluted to 1:50 and phalloidin-TRITC (Sigma-Aldrich) diluted to 1:100 for 1 h at 37 °C. The cell nuclei were stained with DAPI (Vector Laboratories, Inc, Burlingame, CA, USA). Glass coverslip mounting was performed in Vectashield (Vector Laboratories). The immunofluorescence and fluorescence analyses of both the BNFs and BCAFs were carried out on an inverted and motorized microscope (Axio Observer Z.1) equipped with a 63×/1.4 Plan-Apochromat objective. The attached laser-scanning unit (LSM 700 4× pigtailed laser 405-488-555-639; Zeiss, Jena, Germany) enabled confocal imaging. For excitation, 405, 488 and 555 nm lasers were used. Fluorescence emission was revealed by a main dichroic beam splitter and a variable secondary dichroic beam splitter. Triple staining fluorescence images were acquired separately using the ZEN Black 2012 software in the red, green and blue channels at a resolution of 512 × 512 pixels, with the confocal pinhole set to one Airy unit and then saved in the TIFF format. All other chemicals were of analytical grade and were purchased from Sigma-Aldrich.

### 4.3. Cell Viability and Wound-Healing Assays for Human Primary Breast Fibroblast Characterization

To evaluate the influence of the BNFs and BCAFs on MCF-7 cell viability in vitro, 1 × 10^4^ MCF-7 cells/well were seeded in 96-well plates and let to grow for 24 h. Then, the culture medium was removed and replaced with 100 μL of the conditioned media from the BNFs and BCAFs. After 48 h of incubation, an ATP assay was performed by a CellTiter-Glo^®^ Luminescent Cell Viability Assay kit (Promega, Madison, WI, USA), according to the manufacturer’s protocol.

A Wound-Healing Assay was performed to analyze and compare the influence of BNFs and BCAFs on MCF-7 cell migration in vitro. Briefly, 5 × 10^5^ MCF-7 cells were cultured in each well of a 12-well plate and grown until 100% confluence. Then, the confluent monolayer of the cells was carefully scratched with a sterilized pipette tip, and, after wounding, the plates were rinsed with 1 × PBS to remove cell debris. Cells were incubated with the conditioned media from the BNFs and BCAFs. Photographs of the same fields were taken at 0 and 48 h with a camera connected to a CKX41 model Olympus phase-contrast microscope and the images were acquired using the Cell-A software. A quantitative analysis of the open wound was performed by measuring the gap area at different time points using the Image J software, version 1.48i (the National Institute of Health, Bethesda, MD, USA). The ratio between the relative open surface area at the indicated time points and at baseline was calculated.

### 4.4. Real Time PCR

Total RNA was extracted from the cell samples using Trizol reagent (Invitrogen, Carlsbad, CA, USA) or in alternative Maxwell RSC simplyRNA Cells (Promega) and was processed with a Maxwell RSC Instrument (Promega) according to the manufacturer’s instructions. A reverse transcription reaction was performed using the Quantitect Reverse Transcription kit (Qiagen) following the manufacturer’s directions. A quantitative Real-Time PCR (qRT-PCR) was performed in LightCycler 96 (Roche, Penzberg, Germany) using LightCycler FastStart DNA Master SYBR Green I (Roche) and each validated primer. The validated qRT-PCR primers were from Eurofins (Milan). GAPDH was used as an internal control. Total RNA from formalin fixation and paraffin embedding (FFPE) tissues was isolated using the Maxwell RSC RNA FFPE kit (Promega, Madison, WI, USA) and was processed with a Maxwell RSC Instrument (Promega, Madison, WI, USA) as described in the study of Barbato et al. [65]. The primer sequences are listed in Table 2.

### 4.5. Immunohistochemistry

The BCAF spheroids collected at 72 and 216 h were fixed in 10% neutral buffered formalin. Then, each spheroid was embedded in paraffin (Bio-Optica Milano SpA, Milan, Italy), sliced into serial 4 μm-thick sections and placed on poly-l-lysine-coated glass slides (Menzel-Glaser, Brunswick, Germany). The slides were deparaffinized twice in xylene, rehydrated and immersed in 10 mM of citric acid (Sigma-Aldrich), pH 6, in a microwave oven (VWR) for three cycles of 5 min at 650 Watt to exclude epitope masking owing to fixation. The spheroids deparaffinized sections were hematoxylin and eosin stained (Bio-Optica) according to the manufacturer’s protocol. These sections, on glass slides, were converted into high-resolution digital data through NanoZoomer-2.0RS digital scanner (Hamamatsu, Tokyo, Japan, Asia). Other sections were immunostained with primary antibodies against vimentin (Ventana Medical Systems, Tucson, AZ, USA), COX-2 and *α*-SMA (Abcam, Cambridge, UK), detected by the ULTRA View UNIVERSAL DAB DETECTION KIT (Ventana Medical Systems), according to the manufacturer’s protocol.

### 4.6. Generation and Growth of BCAF Spheroids and Cell Culture

The BCAF spheroids were generated using the hanging-drops and agarose-coated U-bottom well plates adapted methods, as previously described [33]. The reverted BCAFs were obtained as previously described [14]. Briefly, the BCAF spheroids collected after 216 h of 3D cell culture were transferred to cell culture plates, expanded and maintained in culture as 2D monolayers for about 20–30 days to allow the full outgrowth of the BCAFS from the spheroids. The BCAFs, grown as a monolayer or as spheroids, and the reverted BCAFs were cultured in DMEM, supplemented with 20% FBS, 200 mM of l-glutamine, penicillin (100 mg/mL) and streptomycin (100 mg/mL) in a humidified incubator at 37 °C under 5% CO_2_ atmosphere. All the experiments were performed only with cells from an early passage (<10).

The MCF-7 BC cell line was kindly provided by CEINGE (Naples, Italy) and cultured at 37 °C in 5% CO_2_ in DMEM, containing 10% FBS, 200 mM of l-glutamine, penicillin (100 mg/mL) and streptomycin (100 mg/mL).

Cell treatments were carried out after 24 h from plating. The cells and spheroids were observed with a phase-contrast microscope Olympus (Tokyo, Japan, Asia) CKX41 model; the images were acquired with a camera connected to the microscope by means of Cell-A software. All other chemicals were of analytical grade and were purchased from Sigma-Aldrich.

### 4.7. Total Cell Lysates and Western Blotting Analysis

The BNFs, BCAFs and spheroids were collected, washed with PBS, lysed in an ice-cold modified RIPA buffer (50 mM of Tris-HCl, pH 7.4, 150 mM of NaCl, 1% Nonidet P-40, 0.25% sodium deoxycholate, 1 mM of Na_3_VO_4_ and 1 mM of NaF) supplemented with Protease Inhibitor Cocktails (Roche Diagnostics Corporation, Mannheim, Germany) and incubated for 30 min on ice. The supernatant, obtained after centrifugation at 12,000× *g* for 30 min at +4 °C, provided the total protein extract. The protein concentration was determined by Bradford’s method [66]. For the western blot analysis, equal amounts of total protein extracts (20 μg) were loaded per lane on a 10% sodium dodecyl sulphate-polyacrylamide gel (SDS-PAGE). Following electrophoresis and a transfer to the Immobilon P membrane (Millipore), the membrane was incubated in 5% non-fat milk in PBS-Tween. The filter was incubated with the specific primary antibody at 4 °C overnight, COX-2, *α*-SMA (Abcam, Cambridge, UK), vimentin (Cell Signal Technology Inc., Danvers, MA, USA), GAPDH (Cell Signal Technology) and then with the horseradish peroxidase-linked secondary antibody at room temperature for 1 h. The membranes were then analyzed by an enhanced chemiluminescence reaction, using a Super Signal West Pico kit (Pierce) according to the manufacturer’s instructions; the signals were visualized by autoradiography.

### 4.8. MTT Assay

The MTT assay was used to detect the proliferation of the MCF-7 cells treated with the conditioned media derived from BCAF monolayers, BCAF spheroids and reverted BCAFs. A standard cell culture medium was used as the control unconditioned medium. Briefly, the MCF-7 cells were seeded on 96-well plates (1 × 10^4^ cells/well) and let to grow for 24 h. Then, the culture medium was removed and replaced with 100 μL of the conditioned media. After 48 h of incubation, 10 μL of 12 mM of MTT solution was added to each well, incubated for 3 h at 37 °C in the dark. Then, the conditioned media were aspirated and the MTT formazan crystals were dissolved in 100 μL of solubilization solution (0.01 M HCl in isopropanol). Cell number is correlated with the amount of formazan crystals formed. Absorbance at 570 nm was measured using a Multi-scan EX microplate reader (Thermo Labsystems, Vantaa, Finland).

All chemicals were of analytical grade and were purchased from Sigma-Aldrich.

### 4.9. Analysis of BC Cell Migration

Cell migration in the 2D culture condition was assessed using a Wound-Healing Assay. The MCF-7 cells (5 × 10 ^5^ cells/well) were cultured in 12-well plates and were grown until 100% confluence. The confluent cells were carefully scratched using a sterilized pipette tip and then washed with 1 X PBS to remove cell debris. MCF-7 cells were incubated with the control medium, the conditioned medium derived from BCAF monolayers or the conditioned medium from reverted BCAFs. Photographs of the same fields were taken at 0 h and 24 h. The images were acquired with a camera connected to a phase-contrast microscope Olympus CKX41 model, using Cell-A software. A quantitative analysis of the open wound was performed by measuring the gap area at different times using ImageJ Software (the National Institute of Health, USA).

### 4.10. Statistical Analysis

Numerical data were reported in Kaleida Graph 4.0 and analyzed by a student’s *t*-test; a one-way ANOVA, with Bonferroni corrections, was used for multiple comparisons.

## 5. Conclusions

The interactions between the TME and cancer cells dramatically influence the development and growth of solid tumors and the outcome of therapeutic strategies. Therefore, understanding if the TME is irreversibly differentiated into a pro-tumor phenotype or if some components of the TME can return to a less pro-tumorigenic or physiological phenotype is of great importance to both improve current anti-cancer therapies and to develop new therapeutic strategies [67].

BCAFs represent the most abundant cell type among non-cancer stromal cells of the breast TME and show a constitutively activated phenotype sustaining cancer growth and progression. In this work, we demonstrated that it is possible to reprogram BCAFs toward a less activated cell type even without drug treatment in vitro and that BCAF inactivation is associated with a decrease in cancer cell viability and migration in vitro. In particular, we demonstrated that the conditioned media from BCAF spheroids and reverted BCAFs hinder the viability and migration of cancer cells, respectively. To the best of our knowledge, this is the first time that BCAFs have acquired an inactivated phenotype without the use of molecules or drug treatment. This work indicated that BCAF inactivation could be a useful tool to develop new therapeutic strategies targeting the interaction between TME and cancer cells. We strongly believe that an improved comprehension of how to appropriately target or modulate the activated phenotype of BCAFs and the interaction between BCAFs and BC cell will be critical to hinder BC progression and dissemination.

## Figures and Tables

**Table 1:** Clinico-pathological parameters of patients (n = 8) with invasive BCs used for BCAF isolation.

Mean Age ± SD (Years)	60.25 ± 6.31
Tumor Grade	
G1	1
G2	2
G3	5
Tumor Stage	
T0	0
T1	3
T2	5
T3	0
T4	0
Lymph Node status	
N0	7
N1	1
ER status	
positive	8
negative	0
PR status	
positive	8
negative	0
HER2 status	
positive	0
negative	8

**Table 2:** Sequence of oligonucleotides used in qRT-PCR analysis.

Gene	Sequence
SPARC	Forward: 5′-TCTTCCCTGTACACTGGCAGTTC-3′ Reverse: 5′-AGCTCGGTGTGGGAGAGGTA-3′
FAP	Forward: 5′- GGAAGTGCCTGTTCCAGCAATG-3′ Reverse: 5′- TGTCTGCCAGTCTTCCCTGAAG-3′

## Data Availability

Not applicable.
